# The Supersulfide-Producing Activity of Rat Cystathionine γ-Lyase Is Irreversibly Inactivated by L-CysNO but Not by L-GSNO

**DOI:** 10.3390/antiox14091113

**Published:** 2025-09-13

**Authors:** Shoma Araki, Tsuyoshi Takata, Sunghyeon Yoon, Shingo Kasamatsu, Hideshi Ihara, Hidehiko Nakagawa, Takaaki Akaike, Yukihiro Tsuchiya, Yasuo Watanabe

**Affiliations:** 1 Department of Pharmacology, Showa Pharmaceutical University, Machida 194-8543, Japan; yakuri.araki@gmail.com (S.A.); yoonsh@ac.shoyaku.ac.jp (S.Y.); 2 Department of Redox Molecular Medicine, Graduate School of Medicine, Tohoku University, Sendai 980-8575, Japan; tsuyoshi.takata.a5@tohoku.ac.jp (T.T.); takaike@med.tohoku.ac.jp (T.A.); 3 Department of Biological Chemistry, Graduate School of Science, Osaka Metropolitan University, Sakai 599-8531, Japan; kasamatsu@omu.ac.jp (S.K.); iharah@omu.ac.jp (H.I.); 4 Graduate School of Pharmaceutical Sciences, Nagoya City University, Nagoya 467-8603, Japan; deco@phar.nagoya-cu.ac.jp

**Keywords:** cystathionine γ-lyase (CSE), supersulfides, nitric oxide (NO), S-nitrosocysteine (CysNO), redox regulation

## Abstract

Cystathionine γ-lyase (CSE) is a pyridoxal 5′-phosphate (PLP)-dependent enzyme that catalyzes the final step of the transsulfuration pathway, converting cystathionine into cysteine. Additionally, CSE is also essential for the formation of cysteine hydropolysulfide (Cys-S-(S)n-H), known as supersulfides, by metabolizing cystine under pathological conditions. We previously reported that, during cystine metabolism, CSE undergoes self-inactivation through polysulfidation at the Cys136 residue. Here, contrary to the anticipated role of L-S-nitrosocysteine (L-CysNO) as a nitric oxide (NO) donor, we demonstrate that it serves as a substrate for CSE and that its metabolites inhibit the activity of the enzyme during L-CysNO metabolism. The in vitro incubation of CSE—but not the Cys136/171Val mutant—with L-CysNO resulted in the dose-dependent inhibition of supersulfide production, which was not reversed by the reducing agents. Notably, CSE activity remained unchanged upon preincubation with other NO donors, such as S-nitrosoglutathione or D-CysNO, but was inhibited when coincubated with cysteine. Furthermore, when PLP was removed from the CSE/L-CysNO premix, L-CysNO no longer inhibited CSE activity, suggesting that CSE metabolizes L-CysNO and that its metabolites contribute to enzyme inactivation. Indeed, we identified thionitrous acid and pyruvate as the primary CSE/L-CysNO reaction products. Thus, we establish L-CysNO as a CSE substrate and demonstrate that its metabolites act as enzyme inhibitors through a novel irreversible modification at the Cys136/171 residues.

## 1. Introduction

**Scheme 1:**
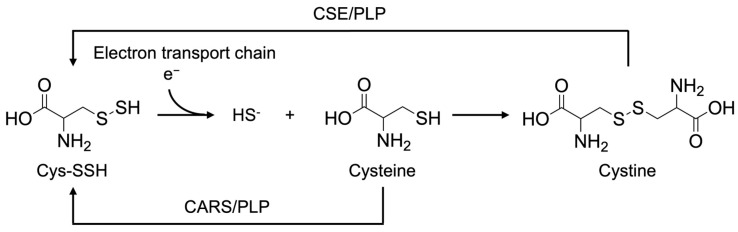
Chemical equation: Cysteine persulfide is generated by CSE and CARS in a PLP-dependent manner.

Supersulfides are sulfur species with catenated sulfur atoms, which include hydropersulfides (RSSHs) and polysulfide species (RSSnR; *n* > 1, R = hydrogen, and alkyl or cyclic sulfurs) [1,2]. They have strong nucleophilic and antioxidant properties, surpassing those of thiols and thereby acting as redox-signaling molecules [3]. They also serve as electron acceptors, with cysteine hydropersulfide being reduced to H_2_S in the mitochondrial electron transport chain. This process is closely associated with energy metabolism and lifespan [3,4]. In mammals, endogenous cysteine hydropersulfide can be synthesized by the pyridoxal 5′-phosphate (PLP)-dependent activity of cysteinyl-tRNA synthetase (CARS) using cysteine as a substrate [3] (Scheme 1). Cysteine is synthesized via the transsulfuration pathway involving the PLP-dependent enzymes cystathionine β-synthase (CBS) and cystathionine γ-lyase (CSE), where CSE catalyzes the conversion of cystathionine, produced by CBS, into cysteine [5,6]. CSE functions as a key enzyme in various tissues under pathophysiological conditions, contributing significantly to the cellular response to oxidative stress [7,8,9,10,11].

CSE has also been reported as another enzyme that generates cysteine hydropersulfide (Cys-SSH) using cystine—instead of cysteine—as a substrate in oxidative stress conditions [1] and produces H_2_S using ergothioneine, a dietary thione/thiol that improves the health and lifespan of aged animals [12]. CSE was upregulated in response to short-term 50% dietary restriction, contributing to oxidative stress resistance under sulfur amino acid restriction in mice [13,14]. Thus, CSE may serve as a simple molecular biomarker associated with longevity [15,16]. CSE can be regulated by post-translational modifications. Specific phosphorylation sites have been identified, resulting in either enhanced or reduced CSE activity under hypoxic conditions in endothelial cells [17] or normoxic conditions in the carotid body [18], respectively. CSE has also been identified as a cysteine-based redox-sensitive enzyme, as the endogenous S-nitrosylation of CSE at Cys136/171 has been reported in mouse liver [19]. In human aortic vascular smooth muscle cells, H_2_O_2_-triggered stable disulfide bond formation between Cys252 and Cys255 enhances the H_2_S-producing activity of CSE [20]. CSE nitration and polysulfidation have also been reported, although the significance of these site-specific modifications has yet to be fully investigated [21,22]. It has also been demonstrated that the physiological NO donor S-nitrosoglutathione (GSNO) inhibits the H_2_S-generating activity of human CSE from cysteine through *S*-nitrosylation at Cys137 (equivalent to Cys136 in mice and rats) [23]. We recently reported that CSE self-inactivates through polysulfidation at the Cys136 residue during cystine metabolism [24].

Therefore, our initial objective was to determine whether GSNO can directly affect the cysteine hydropersulfide-producing activity of CSE, given that cysteine hydropersulfide can be synthesized by CSE independently of H_2_S [1]. Experiments were also performed to analyze whether L-S-nitrosocysteine (L-CysNO) affects the cysteine hydropersulfide-producing activity of CSE in the presence or absence of PLP, since L-CysNO—but not GSNO—altered this activity. We propose that L-CysNO is an endogenous CSE substrate and suggest that the reaction products, rather than acting as NO donors, are the main factors responsible for decreased CSE hydropersulfide-producing activity during L-CysNO metabolism.

## 2. Materials and Methods

### 2.1. Materials

The cDNA for rat CSE (pME18S-ratCSE-HA) was a generous gift from Dr. Nozomu Nishi [25] (Life Science Research Center, Kagawa University, Kagawa, Japan). The anti-CSE antibody was prepared as described previously [26]. Sulfane sulfur probe 4 (SSP4) was obtained from Dojindo laboratories (Kumamoto, Japan). All other reagents and materials were obtained from commercial sources and were of the highest available quality.

### 2.2. Plasmid Used

The cDNA of rat CSE was amplified by PCR using gene-specific primers containing XhoI and NotI restriction sites against the pME18S-ratCSE-HA plasmid. The primers used are listed in the Supplementary Information (Table S1). The PCR product and the recipient vector (pGEX-6P) were digested with XhoI and NotI and ligated to generate an Escherichia coli expression plasmid of pGEX-ratCSE-HA. The CSE mutants C136V, C171V, and C136V/C171V (with both Cys136 and Cys171 substituted by valine) were generated using pME18S or pGEX plasmid DNA and a QuickChange II Site-Directed Mutagenesis Kit (Agilent Technologies, Inc., Santa Clara, CA, USA). The primers used are listed in the Supplementary Information (Table S1). The nucleotide sequences of all constructs were confirmed by sequencing analysis.

### 2.3. CSE Purification

Recombinant rat GST-CSE was expressed in *E. coli* (DH5α) and purified using glutathione affinity chromatography. Briefly, cell lysates were applied to a GSH-agarose column (GSH accept, Nacalai Tesque, Kyoto, Japan) [27], and the bound GST-CSE was treated on-column with Turbo3C Protease (Accelagen, Inc., San Diego, CA, USA) to cleave the GST tag [28]. The flow-through containing purified CSE was collected, concentrated, and used for subsequent experiments. Protein levels were measured using the Bradford assay with BSA as the standard [29].

### 2.4. Measurement of CSE Activity

The Cys-SSH produced by CSE with cystine as the substrate was measured by using SSP4, a fluorescent probe specific for sulfane sulfurs [24]. We first established an enzyme titration curve and time course for the SSP4 assay, using 1 mM cystine as the substrate. Reaction rates were calculated from the slope of the linear portion of the curve. Accordingly, an enzyme concentration of 50 µg/mL in a 20 µL reaction mixture with a 20 min incubation was chosen for the assay. Recombinant CSE (50 µg/mL, 1.1 µM) was incubated in 20 µL of 50 mM Hepes buffer (pH 7.5) containing 50 µM PLP, either with buffer alone or 1 mM cystine, for 20 min at 37 °C. Samples were diluted five-fold and incubated with 10 µM SSP4 in 100 µL of 20 mM Tris-HCl buffer (pH 7.4) containing 1 mM cetyltrimethylammonium bromide, in the dark, for 10 min at room temperature. Fluorescence intensity was measured with a microplate reader (Synergy HTX Multimode Reader, BioTek/Agilent Technologies, Inc., Santa Clara, CA, USA) using excitation at 485/20 nm and emission at 528/20 nm. Because DTT and TCEP degrade the Cys-SSH generated by CSE into cysteine, these reducing agents had to be removed. To address this, we developed an immobilized CSE assay. GST-CSE was expressed in E. coli (DH5α) using the pGEX-6P vector and immobilized on GSH-agarose (Supplementary Figure S1). Following treatment with L/D-CysNO or Na_2_S_4_, the reducing agents were eliminated by centrifugation. The immobilized CSE was then washed three times with 50 mM Hepes (pH 7.5) and incubated with 50 µM PLP and 1 mM cystine at 37 °C for 20 min. The resulting Cys-SSH levels were quantified as described above. The cysteine produced by CSE from cystathionine was measured by DTNB assay [30] using immobilized GST-CSE. CSE activity was measured in 40 mM borate buffer (pH 8.2) containing 1 mM cystathionine, 50 µM PLP, and 1 mM DTNB at 30 °C for 10 min. The reaction was monitored by recording the increase in absorbance at 412 nm (Synergy HTX Multimode Reader, BioTek/Agilent Technologies, Inc., Santa Clara, CA, USA), which reflects the generation of the nitrobenzoate thiolate anion.

### 2.5. HSNO Measurement Using TAP-1

HSNO formation by CSE using CysNO as the substrate was quantified with TAP-1 [31]. Reactions were carried out with 1 mM CysNO, where recombinant CSE (50 µg/mL, 1.1 µM) was incubated in 20 µL of 50 mM Hepes buffer (pH 7.5) containing 50 µM PLP, either with buffer alone or 1 mM cystine, for 20 min at 37 °C. The reaction mixtures were then diluted five-fold and incubated with 10 µM TAP-1 in 100 µL of 20 mM Tris-HCl (pH 7.4) containing 1 mM cetyltrimethylammonium bromide, in the dark, for 10 min at room temperature. Fluorescence was measured with a microplate reader (Synergy HTX Multimode Reader, BioTek/Agilent Technologies, Inc., Santa Clara, CA, USA) using excitation at 485/20 nm and emission at 528/20 nm.

### 2.6. Fluorometric Determination of Pyruvate

Pyruvate oxidase was employed to catalyze the oxidation of pyruvate, generating hydrogen peroxide that subsequently interacted with a fluorescent probe. The measurement was carried out using an EnzyChrom Pyruvate Assay Kit (BioAssay Systems, Hayward, CA, USA) [32,33,34,35,36,37,38]. Recombinant CSEs (50 µg/mL, 1.1 μM) were incubated in 20 μL of 50 mM Hepes (pH 7.5), containing 50 µM PLP with buffer alone or 1 mM cystine or L-CysNO or D-CysNO for 20 min at 37 °C. Samples were diluted five-fold and incubated for 10 min at room temperature in 100 µL of working reagent, following the manufacturer’s instructions. Fluorescence was measured with a microplate reader (Synergy HTX Multimode Reader, BioTek/Agilent Technologies, Inc., Santa Clara, CA, USA) using excitation at 530/20 nm and emission at 585/20 nm.

### 2.7. Live-Cell Fluorescence Imaging of Cys-SSH

COS-7 cells were maintained in Dulbecco’s Modified Eagle Medium (DMEM) supplemented with 10% fetal calf serum (FBS) and 1% penicillin–streptomycin under humidified conditions at 37 °C. For experiments, cells were seeded onto 6 cm dishes at a density of 5 × 10^5^ cells per dish. Transient transfection was carried out using Lipofectamine 3000 (Thermo Fisher Scientific, Waltham, MA, USA). In brief, 1 µg of wild-type, C136V, 171V, or C136V/C171V pME18S-ratCSE-HA plasmid DNA was mixed with 2 µL of P3000 reagent and 3 µL of Lipofectamine 3000 in Opti-MEM, which was then added to the cells. After 24 h of incubation, transfected COS-7 cells were reseeded for imaging and protein analysis: 5 × 10^4^ cells per well in an eight-well chamber slide (µ-Slide 8 Well High, ibidi GmbH, Gräfelfing, Germany) for microscopy and 5 × 10^5^ cells per 6 cm dish for Western blotting. Following an additional 48 h incubation, the cells were rinsed once with serum-free DMEM, incubated for 15 min at 37 °C with 20 µM SSP4 in serum-free DMEM containing 500 µM cetyltrimethylammonium bromide, then counterstained with Hoechst 33342. Excess probe was removed by washing the cells with HBSS. Fluorescence images were acquired with a Nikon A1R+ confocal laser scanning system (Nikon, Tokyo, Japan) mounted on an ECLIPSE Ti-E inverted microscope using a 20× objective lens. Excitation was performed at 488 nm, and emission was collected at 500/50 nm. Fluorescence intensities were analyzed with NIS-Elements imaging software (Ar, version 6.10.00, Nikon, Tokyo, Japan).

## 3. Results

### 3.1. L-CysNO, but Not Other NO Donors, Inhibits CSE’s Cysteine Hydropersulfide-Producing Activity

**Figure 1:**
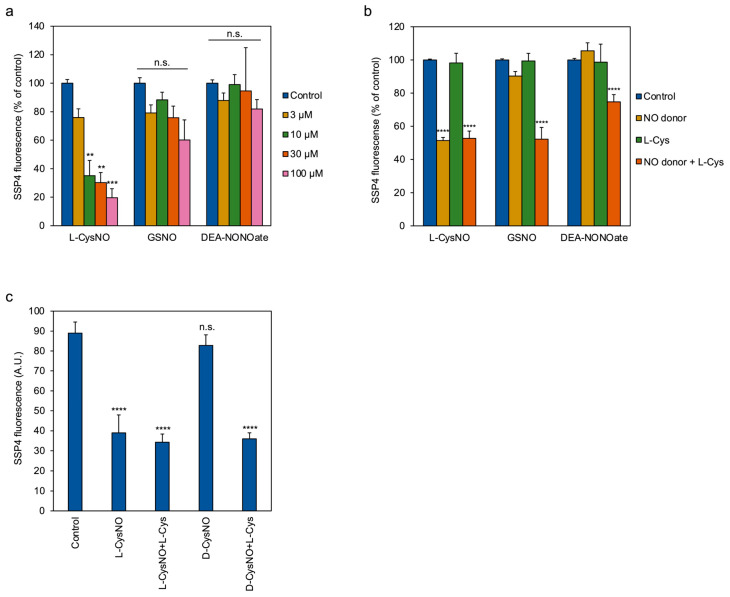
Effect of NO donors on CSE activity. (**a**) Concentration-dependent inhibition of CSE activity by incubation with L-CysNO. Purified CSE (50 µg/mL) was incubated in 20 μL of a solution at 30 °C for 30 min with varying concentrations of NO donor compounds in a buffer containing 50 mM Hepes/NaOH (pH 7.5) and 50 µM PLP. Enzyme activity was subsequently measured by adding 1 mM cystine, and the resulting Cys-SSH levels were quantified using the SSP4 probe. (**b**,**c**) Effect of cysteine on NO donor-mediated modulation of CSE activity. Purified CSE (50 µg/mL) was incubated in 20 μL of a solution at 30 °C for 30 min with 10 µM NO donor compounds in the same buffer, in the presence or absence of 10 µM cysteine. Results are the mean ± SE of four independent experiments. **** *p* < 0.0001, *** *p* < 0.001, and ** *p* < 0.01, unless otherwise indicated in the figure, compared with the controls of each sample. n.s.: not significant.

It was shown that CSE-mediated H_2_S production was inhibited by either diethylamine (DEA)-NONOate or GSNO when cysteine was used as a substrate [23,39]. Therefore, we first evaluated the ability of these NO-donors, along with L-CysNO, to inhibit the cysteine hydropersulfide-producing activity of CSE; this was achieved using cystine as a substrate and a sulfur-specific fluorescent probe, SSP4. When we tested the effect of various concentrations of L-CysNO on CSE activity, suppression was shown to occur in a dose-dependent manner, with an apparent IC_50_ value of 10 µM (Figure 1a). Although DEA-NONOate or GSNO alone did not inhibit CSE activity, the coincubation of these NO-donors with L-cysteine resulted in attenuated CSE activity (Figure 1b). Next, we determined whether D-CysNO, a stereoisomer of L-CysNO, could also inhibit CSE activity. Although equal amounts of D-CysNO and L-CysNO produced a similar amount of NO, L-CysNO alone inhibited CSE activity, whereas D-CysNO inhibited it only in the presence of L-Cys (Figure 1c).

**Figure 2:**
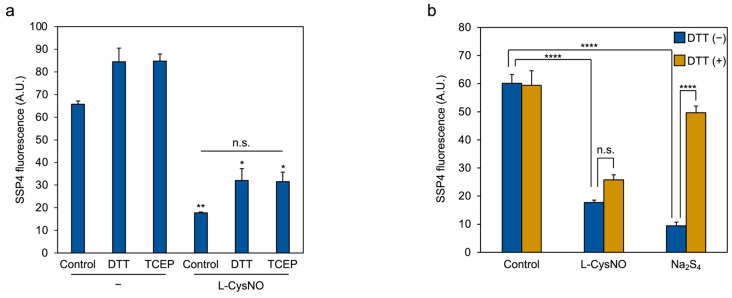
Effect of DTT and TCEP on the reversibility of CSE inhibition by either L-CysNO or Na_2_S_4_. Immobilized GST-CSE was preincubated with either 100 μM L-CysNO (**a**,**b**) or 10 μM Na_2_S_4_ (**b**) in the presence of 50 µM PLP at 30 °C for 30 min. After centrifugation to remove residual L-CysNO or Na_2_S_4_, CSE activity was assessed in a reaction mixture containing 50 µM PLP and 1 mM cystine. The production of Cys-SSH was then detected using the SSP4 fluorescent probe. For the DTT (**a**,**b**) and TCEP (**a**) conditions, samples were additionally incubated with 10 mM DTT and 10 mM TCEP at 30 °C for 10 min, followed by centrifugation prior to assessment. Data are shown as mean ± SE of four independent experiments. Significance versus the respective controls is indicated as **** *p* < 0.0001, ** *p* < 0.01, and * *p* < 0.05; n.s., not significant.

Since protein polysulfidation has been described as reversible post-translational modifications, we examined whether the inhibition of CSE by L-CysNO could be reversed by treatment with reducing agents. To this end, immobilized CSE was preincubated with L-CysNO, followed by the addition of either thiol-containing or thiol-free reducing agents. As shown in Figure 2a, L-CysNO-induced inhibition of CSE activity was not restored by the subsequent addition of the thiol-containing reducing agent dithiothreitol (DTT) or the thiol-free reducing agent tris(2-carboxyethyl)phosphine (TCEP). In contrast, and consistent with our earlier study, pretreatment of immobilized CSE with 10 μM Na_2_S_4_ suppressed its β-lyase activity toward cystine through polysulfidation, and this inhibition was completely reversed upon DTT addition (Figure 2b). Taken together, these findings indicate that, unlike polysulfidation, L-CysNO-mediated modification leads to the irreversible inhibition of CSE. Moreover, the results suggest that the formation of L-CysNO itself, rather than the presence of NO donors alone, is required to achieve this irreversible suppression of CSE activity when cystine is used as the substrate.

### 3.2. CSE Activity Inhibition by L-CysNO Requires the Presence of PLP

**Figure 3:**
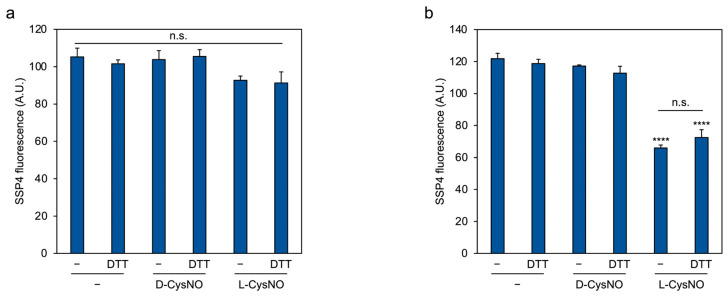
Effect of PLP on L-CysNO-induced inhibition of CSE activity. Immobilized GST-CSE was preincubated with 100 μM D- or L-CysNO at 30 °C for 30 min, either without (**a**) or with (**b**) 50 µM PLP. CSE enzymatic activity in each reaction mixture was assessed with 50 µM PLP and 1 mM cystine, according to the procedure outlined in Figure 2. Data are presented as mean ± SE from four independent experiments. Statistical significance versus the respective controls is indicated as **** *p* < 0.0001; n.s., not significant, unless noted otherwise in the figure.

The above data suggest a causal role of L-CysNO-derived metabolites in the subsequent inhibition of CSE activity. To further investigate this concept, we examined whether L-CysNO could inhibit this activity in the absence of PLP, which is an essential cofactor for CSE. CSE pretreatment with L-CysNO in the absence of PLP failed to inhibit enzyme activity (Figure 3), indicating that CSE metabolizes L-CysNO and its metabolites inactivate the enzyme.

**Scheme 2:**
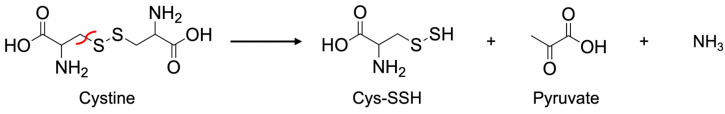
CSE β-lyase activity toward cystine in generating Cys-SSH. The red line indicates the position at which CSE exhibits C-S lyase activity toward cystine.

**Scheme 3:**
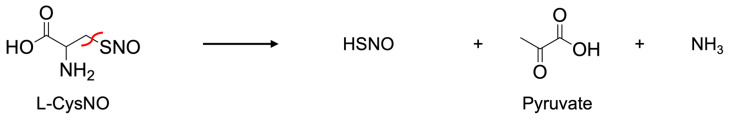
CSE β-lyase activity toward L-CysNO in generating HSNO. The red line indicates the position at which CSE exhibits C-S lyase activity toward L-CysNO.

Since CSE shows β-lyase activity toward cystine—an oxidized derivative of Cys—in producing cysteine hydropersulfide and pyruvate (Scheme 2), it could also exhibit β-lyase activity toward L-CysNO, producing thionitrous acid (HSNO) and pyruvate as the primary products (Scheme 3).

**Figure 4:**
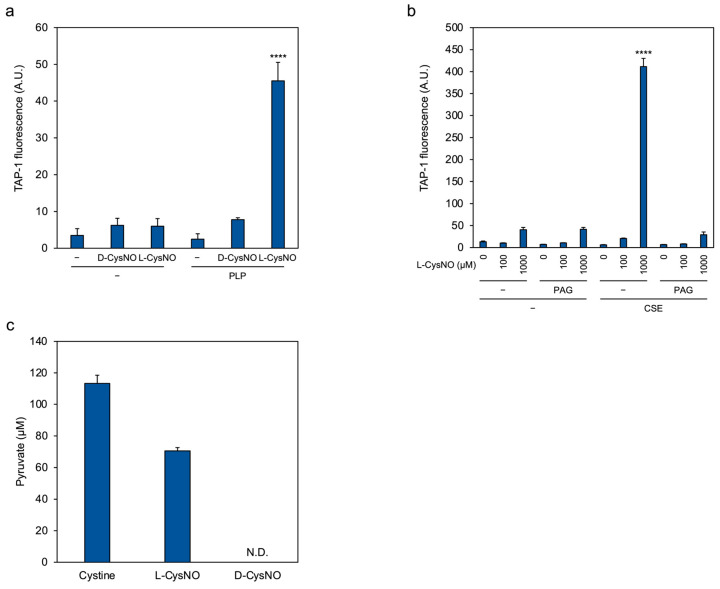
Product analysis of CSE with L-CysNO. (**a**,**b**) HSNO production. Purified CSE (50 µg/mL) was incubated in 20 μL of a solution at 30 °C for 30 min with 100 μM D- or L-CysNO, either without or with 50 µM PLP (**a**) or with the indicated concentrations of L-CysNO and 50 µM PLP in the absence or presence of 1 mM PAG (**b**) in a buffer containing 50 mM Hepes/NaOH (pH 7.5) and 50 µM PLP. HSNO levels were quantified using the TAP-1 probe. (**c**) Pyruvate production. Purified CSE (50 µg/mL) was incubated in 20 μL of a solution at 30 °C for 30 min with 1 mM cystine or D- or L-CysNO in the presence of 50 µM PLP in the same buffer. Pyruvate production was measured using a fluorescent probe specific for hydrogen peroxide. Results are the mean ± SE of four independent experiments. **** *p* < 0.0001 when compared with the controls of each sample.

We analyzed CSE/L-CysNO reaction products using the fluorescent probe TAP-1 for HSNO detection [31]; we also used a fluorescent probe for detecting the hydrogen peroxide produced by pyruvate/pyruvate oxidase in order to quantify pyruvate, following the manufacturer’s instructions. The PLP-dependent β-lyase activity of CSE toward L-CysNO—but not D-CysNO—in producing HSNO was evident, and this was dampened by DL-propargylglycine (PAG) pretreatment, a CSE inhibitor (Figure 4a,b). Pyruvate production was also observed when CSE was treated with L-CysNO but not with D-CysNO (Figure 4c).

**Figure 5:**
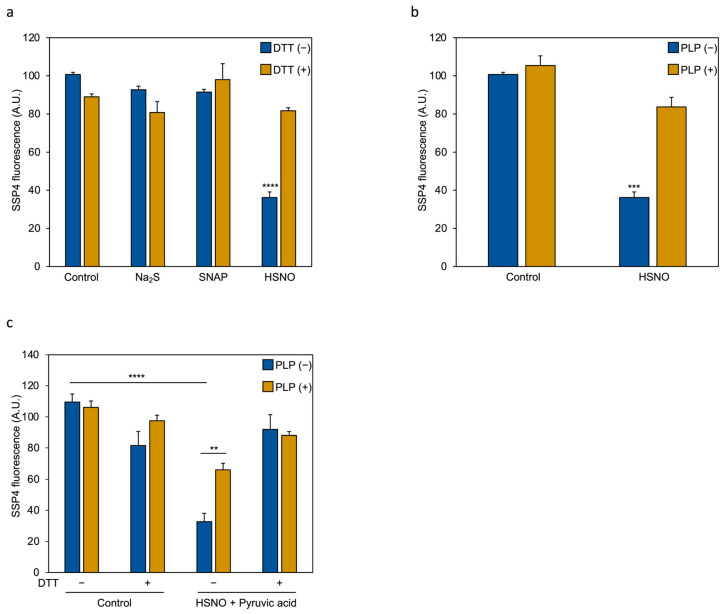
Effect of HSNO source on CSE activity. (**a**) Immobilized GST-CSE was preincubated with 100 μM Na_2_S, 100 μM SNAP, or 100 μM HSNO source at 30 °C for 30 min without 50 µM PLP. (**b**) Immobilized GST-CSE was preincubated with 100 μM HSNO source at 30 °C for 30 min, either without (PLP−) or with (PLP+) 50 µM PLP. CSE activity in each reaction mixture was measured with 50 µM PLP and 1 mM cystine, following the method shown in Figure 2. (**c**) Immobilized GST-CSE was preincubated with buffer alone (Control) or 100 μM HSNO source and 100 μM pyruvic acid at 30 °C for 10 min, either without (PLP−) or with (PLP+) 50 µM PLP. The enzymatic activity of CSE in each reaction mixture was then measured in the presence of 50 µM PLP and 1 mM cystine, following the procedure described in Figure 2. Data are shown as mean ± SE of four independent experiments. Significance versus control or indicated column: **** *p* < 0.0001, *** *p* < 0.001, and ** *p* < 0.01.

These results suggest that HSNO and pyruvate are the primary products of the CSE/L-CysNO reaction, and these metabolites might be involved in irreversible CSE inhibition. HSNO is formed through the reaction of H_2_S with RSNOs or NO [40,41,42,43,44,45,46,47,48,49,50,51,52,53]. To generate HSNO, we freshly prepared a mixture of 1 mM S-nitroso-N-acetylpenicillamine (SNAP, a NO donor) and 0.3 mM Na_2_S in 50 mM Tris-HCl buffer (pH 7.4). This mixture was confirmed to react with TAP-1 and was subsequently used as the HSNO source. Treatment of CSE with a one-third dilution of the HSNO source (100 μM), but not with 100 μM SNAP or 100 μM Na_2_S alone, resulted in inhibition of its enzymatic activity, which was restored upon the addition of DTT (Figure 5a). Notably, this inhibition was not observed when PLP was included in the reaction mixture (Figure 5b). Furthermore, coincubation of pyruvate with the HSNO source led to inhibition of CSE activity, comparable to that observed with the HSNO source alone.

### 3.3. Cys136 and/or Cys171 Are CSE Sensors of L-CysNO-Induced Enzyme Inhibition

**Figure 6:**
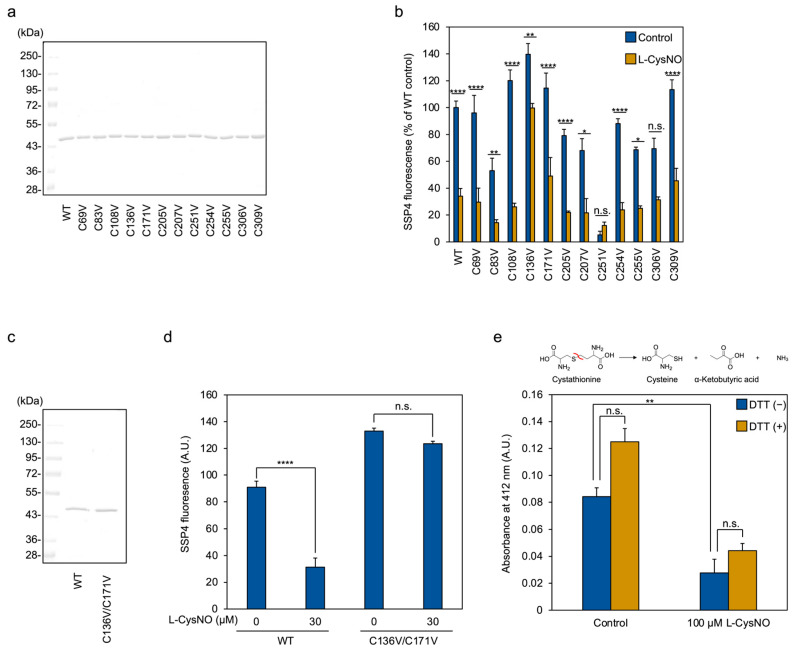
Cysteine residues involved in L-CysNO-induced CSE activity inhibition. (**a**) Preparation of purified recombinant CSEs used in this study. Equivalent amounts (1 µg) of GST-cleaved wild-type CSE (WT) and the specified mutants, expressed in *E. coli*, were resolved on 10% SDS-PAGE and visualized with Coomassie Brilliant Blue. (**b**) Purified wild-type CSE and the indicated cysteine-to-valine mutants (50 µg/mL) were incubated at 30 °C for 10 min either in the absence or presence of 100 µM L-CysNO and 50 µM PLP. Enzyme activity was assessed by adding 1 mM cystine, following the protocol described in Figure 1a. (**c**) Equal amounts (0.5 µg) of purified WT and the C136V/C171V double mutant expressed in E. coli were analyzed by 10% SDS-PAGE and stained with Coomassie Brilliant Blue. (**d**) Purified WT and the indicated CSE mutants (50 µg/mL) were incubated at 30 °C for 10 min with or without 30 µM L-CysNO and 50 µM PLP. Enzyme activity was then evaluated by introducing 1 mM cystine, as described in Figure 1a. (**e**) Effect of L-CysNO on the γ-lyase activity of CSE toward cystathionine in generating cysteine (see inset). Immobilized GST-CSE was preincubated with 100 µM L-CysNO and 50 µM PLP at 30 °C for 10 min. After removing residual L-CysNO by centrifugation, CSE activity was measured in 50 µM PLP, 1 mM DTNB, and 1 mM cystathionine at 37 °C for 20 min, with absorbance at 412 nm. For DTT treatment, samples were incubated with 10 mM DTT prior to measurement. Data are mean ± SE of four independent experiments. Significance versus control: **** *p* < 0.0001, ** *p* < 0.01, and * *p* < 0.05; n.s., not significant.

We previously reported that Cys136 is a CSE redox sensor during β-lyase activity toward cystine, generating cysteine hydropersulfide [24]. In human CSE, the Cys137 residue (corresponding to Cys136 in mice and rats) has been found to play a key role in S-nitrosoglutathione-induced CSE activity inhibition in producing H_2_S from cysteine [23]. Therefore, we tested whether Cys136 is a critical residue for L-CysNO-induced CSE inhibition. The rat CSE enzyme comprises twelve cysteine residues. To identify which of these are susceptible to CysNO-induced CSE inactivation, we created rat CSE mutants in which each cysteine was individually substituted with valine. Rat CSE was expressed with a GST tag in pGEX-6P vectors, followed by site-specific GST removal and purification. All purified CSE variants achieved at least 90% purity, displaying a primary band at approximately 45 kDa on SDS-PAGE, visualized with Coomassie Brilliant Blue staining (Figure 6a). The activity of the wild-type enzyme was suppressed upon exposure to 100 µM L-CysNO. In contrast, the CSE mutant C136V exhibited partial resistance to L-CysNO. Other CSE mutants, including C69V, C83V, C108V, C171V, C205V, C207V, C251V, C254V, C255V, C306V, and C309V, showed no significant changes (Figure 6b). Studies have indicated that endogenous CSE undergoes S-nitrosylation at Cys171 and Cys136/Cys171 in the kidney and liver of wild-type mice, respectively [19]. Therefore, a double-mutant CSE (C136V/C171V) was expressed and purified using identical pGEX-6P vectors. The recombinant protein achieved a purity level exceeding 90% and appeared as a prominent 45 kDa band on SDS-PAGE following Coomassie Brilliant Blue staining (Figure 6c). Notably, the C136V/C171V CSE mutant was unaffected by L-CysNO, whereas the wild-type enzyme displayed significant inactivation (Figure 6d). We examined whether the γ-lyase activity of CSE toward cystathionine, which generates cysteine, could also be inhibited by L-CysNO. Preincubation with 100 µM L-CysNO resulted in wild-type enzyme inhibition which was not reversed upon treatment with DTT (Figure 6e).

**Figure 7:**
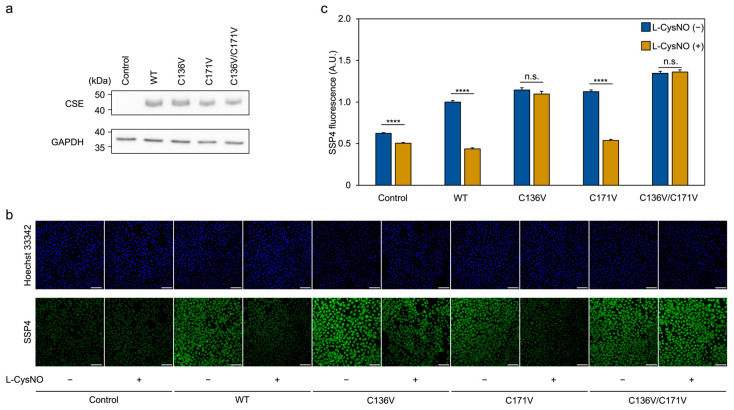
CSE β-lyase activity toward cystine in cells. (**a**) COS-7 cells were transfected with wild-type (WT) CSE cDNA, the indicated CSE mutant cDNAs, or an empty vector (Control) and subjected to Western blot analysis using anti-CSE and anti-GAPDH antibodies. (**b**) Live-cell fluorescence imaging of Cys-SSH production was performed using SSP4 in COS-7 cells transfected with either WT CSE or the indicated mutants in the presence (L–CysNO+) or absence (L–CysNO−) of L-CysNO. Scale bar = 100 µm. (**c**) Relative Cys-SSH levels were quantified based on SSP4 fluorescence intensity in each sample shown in panel (**b**). Data are mean ± SE. **** *p* < 0.0001 versus control; n.s., not significant.

We previously demonstrated that sulfane sulfur levels increased in CSE-overexpressing cells, as detected using the fluorescence probe SSP4 [24]. For further investigation, we assessed β-lyase activity toward cystine in producing Cys-SSH in COS-7 cells expressing either wild-type CSE or its variants (C136V, C171V, or C136V/C171V). Western blot analysis using an anti-CSE antibody (Figure 7a) confirmed that all CSE-overexpressing cells exhibited comparable CSE protein levels. A significant increase in SSP4 fluorescence was observed in COS-7 cells expressing wild-type or mutant CSEs compared to those transfected with the empty vector (Figure 7b,c). Treating cells expressing wild-type or C171V CSE with L-CysNO (100 µM) led to reduced enzyme activity. In contrast, the C136V and C136V/C171V CSE mutants exhibited near-complete resistance to L-CysNO-induced inactivation (Figure 7b,c). Although the endogenous CSE expression level was negligible compared to that of overexpressed rat CSEs, a detectable SSP4 fluorescence signal was observed in empty vector-transfected cells. This fluorescence was reduced upon L-CysNO treatment, suggesting the lower immunoreactivity of the antibody used in this study toward monkey-derived CSE.

**Figure 8:**
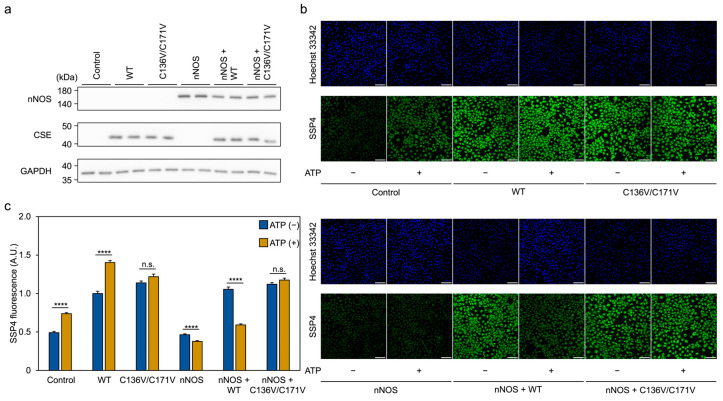
Effect of ATP-induced NOS activation on CSE β-lyase activity in cells. (**a**) Wild-type (CSE) or mutant (C136V/C171V) CSE cDNA was co-transfected into COS-7 cells along with either an empty vector (nNOS−) or nNOS cDNA (nNOS+) followed by Western blot analysis using anti-CSE and anti-nNOS antibodies. (**b**) Fluorescence imaging of Cys-SSH production in live cells. Live COS-7 cells co-transfected with the indicated cDNAs were stained with SSP4 to monitor Cys-SSH production, with or without ATP stimulation. Scale bar = 100 µm. (**c**) Relative Cys-SSH levels were quantified based on SSP4 fluorescence intensity in each sample shown in panel (**b**). Data are presented as mean ± SE. ****p < 0.0001 vs. control, as indicated; n.s., not significant.

To further examine the effect of NOS-derived NO on cellular CSE β-lyase activity, wild-type or C136V/C171V mutant CSE was co-expressed with nNOS in COS-7 cells. β-lyase activity was then evaluated following ATP stimulation, which elevates intracellular Ca^2+^ levels through P2X purinergic receptor activation. Immunoblot analysis confirmed that nNOS and CSE (wild-type or C136V/C171V) were expressed at comparable levels under all conditions (Figure 8a). The overexpression of either wild-type or C136V/C171V CSE alone significantly increased SSP4 fluorescence, with ATP stimulation further enhancing fluorescence in wild-type CSE-expressing cells (Figure 8b,c). When co-expressed with nNOS, wild-type CSE overexpression led to an increase in SSP4 fluorescence, which was subsequently diminished by ATP stimulation. In contrast, C136V/C171V CSE overexpression exhibited a similar increase in SSP4 fluorescence to that of wild-type CSE, but this remained unchanged upon ATP stimulation (Figure 8b,c). These findings suggest that NOS-derived NO is converted to L-CysNO, which acts as a substrate for CSE. The resulting metabolites suppress the cysteine hydropersulfide-producing activity of CSE through modifications at Cys136 and/or Cys171 within cells.

## 4. Discussion

To the best of our knowledge, this is the first study to identify L-CysNO as a substrate for CSE. Previous reports have shown that NO donors inhibit the H_2_S-producing activity of CSE via S-nitrosylation at Cys136 [23,39]. However, our results indicate that NO donors—excluding L-CysNO—do not inhibit cysteine hydropersulfide-producing activity through modification at Cys136 and/or Cys171. This discrepancy is likely due to differences in the experimental substrates used, namely cysteine versus cystine. Since other NO donors can suppress this activity only when coincubated with L-cysteine (Figure 1b), it is plausible that their inhibitory effects are primarily mediated through L-CysNO formation. Although protein S-nitrosylation is generally considered to be fully reversible by reducing agents, Fernandes et al. reported that, when L-cysteine is used as a substrate, the GSNO-induced modification of CSE results in only partial inhibition reversal [23]. In our current study, we found that the L-CysNO-induced inactivation of CSE’s cysteine hydropersulfide-producing activity is not reversed by either thiol-containing or -free reducing agents (Figure 2). Since L-CysNO can be formed through the reaction between GSNO and L-cysteine, these findings suggest that GSNO-induced CSE modification in the presence of L-cysteine may interfere with CSE activity through a mechanism similar to that involved in the CSE/L-CysNO reaction.

Furthermore, L-CysNO-induced CSE inhibition was evident in the presence of PLP (Figure 3), suggesting that CSE/L-CysNO metabolites contribute to CSE activity suppression. Immobilized CSE showed strong, irreversible inhibition when exposed to the reaction products of CSE and L-CysNO. However, since any residual L-CysNO in the reaction mixture is expected to undergo further metabolism by CSE, we investigated whether its metabolites could also inhibit Ca^2+^/calmodulin (CaM)-dependent protein kinase II (CaMKII), a redox-sensitive enzyme regulated via cysteine thiols [54,55]. As shown in Supplementary Figure S2, L-CysNO alone inhibited CaMKII activity, likely via S-nitrosylation, and this inhibition was fully reversed by DTT, consistent with previous findings [55]. Importantly, its inhibition becomes stronger and irreversible when CSE is co-treated with L-CysNO, indicating that CSE metabolizes L-CysNO and that its metabolites inactivate CaMKII through a novel modification, in addition to S-nitrosylation.

What are the essential metabolites responsible for the irreversible inhibition of CSE by L-CysNO? We identified pyruvate and HSNO as initial products of the CSE/L-CysNO reaction (Figure 4). However, the HSNO source, either alone or in combination with pyruvate, was not able to reproduce the PLP-dependent and irreversible inhibition of CSE activity (Figure 5). Furthermore, when PLP was present in the premix, this inhibition was not obvious. The HSNO is expected to exhibit lower stability in solution compared to other RSNO compounds [43]. In vivo quantification remains elusive, and HSNO is widely considered a fleeting intermediate, with its steady-state cellular level—if present at all in aqueous conditions—anticipated to be extremely low [40,41,53,56,57]. Thus, the 100 μM HSNO source used in this study was thought to be pharmacological or pathological rather than physiological. It was shown that HSNO induced the nitrosation of hemoglobin [40]. Membrane-permeable HSNO transfers its “NO+” moiety to protein thiols, generating S-nitrosothiols [41]. Enzymatic modifications mediated by HSNO can regulate essential signaling pathways, particularly those involving redox-sensitive or thiol-containing proteins. To further characterize the reaction products, we utilized SSP4 for sulfane sulfur detection and the fluorescent probe P-Rhod for HNO detection [58]. The β-lyase activity of CSE toward L-CysNO, leading to sulfane sulfur generation, was evident; it was suppressed by pretreatment with PAG, a CSE inhibitor (Supplementary Figure S3a). Additionally, HNO production was detected upon CSE treatment with L-CysNO but not with D-CysNO (Supplementary Figure S3b). These findings suggest that sulfane sulfur and HNO, in addition to HSNO, are metabolic products of the CSE/L-CysNO reaction. We previously demonstrated that sulfane sulfur compounds reversibly inhibit CSE’s cysteine hydropersulfide-producing activity [24]. To test HNO’s role in the observed inhibition, CSE activity was measured in the presence of Angeli’s salt, an HNO donor [59,60,61]. Angeli’s salt inhibited this CSE activity, and like the effect of Na_2_S_4_, this inhibition was reversed in the presence of thiols (Supplementary Figure S4). Furthermore, when PLP was present in the premix, this inhibition was not obvious, even in the effect of Na_2_S_4_. These findings suggest that the detected products—pyruvate, HSNO, sulfane sulfur, and HNO—could not mimic the effects of CSE/L-CysNO metabolites on irreversible CSE inhibition. As we noted, the reaction products of CSE and L-CysNO in the presence of PLP exhibited a significant inhibitory and irreversible effect on both CSE and Ca^2+^/CaM-dependent protein kinase II activities. Thus, irreversible CSE inhibition could occur during L-CysNO metabolism.

To elucidate the specific inhibitory modifications at Cys136 and/or Cys171, we are currently performing a mass spectrometric analysis of synthetic peptides corresponding to CSE 127-145 (FGLKISFVDC136SKTKLLEAA: 2070 Da) and CSE 162-180 (TLKLADIKAC171AQIVHKHKD: 2132 Da) following exposure to CSE/L-CysNO reaction products. Preliminary results indicate the presence of mass spectra peaks at 2131 (2070 + 61) Da and 2161 (2132 + 29) Da, potentially corresponding to CSE127-145-SSNO (SN(S)O, [51]) and CSE162-180-SNO, respectively. Further studies are necessary to confirm these findings.

## 5. Conclusions

**Figure 9:**
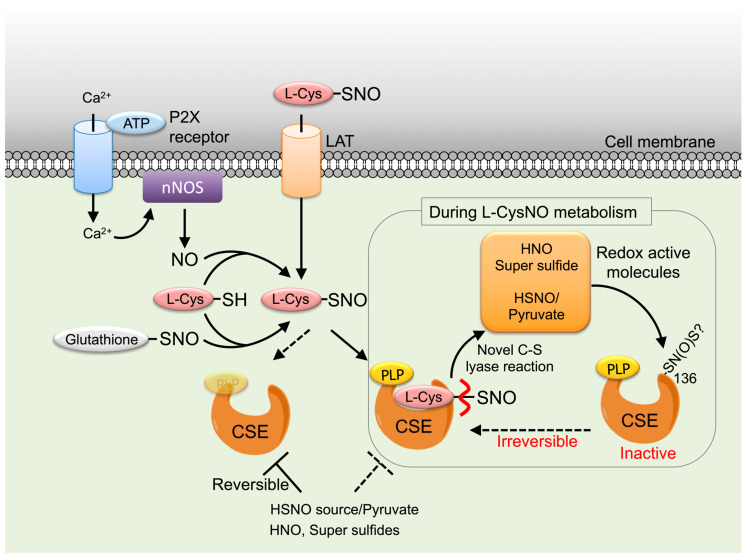
Schematic model of the regulatory interplay between CSE and NOS. ATP-stimulated intracellular Ca^2+^ signaling activates nNOS, leading to NO production in cells co-expressing nNOS and CSE. L-Cysteine reacts with either nNOS-derived NO or S-nitrosoglutathione, generating L-CysNO. In addition, L-CysNO can be transported from the extracellular space via the L-type amino acid transporter (LAT). Subsequently, L-CysNO is metabolized by CSE in a PLP-dependent manner. During this process, CSE activity is irreversibly inhibited through an as yet unidentified modification (possibly –SN(O)S) at Cys136. In contrast, HSNO source, generated from chemical reactions, or HNO/supersulfide donors, exerts a reversible inhibitory effect on CSE in the absence of PLP, but fails to recapitulate the irreversible inhibition induced by L-CysNO.

Our results highlight the critical role of CSE, a pro-longevity gene product [16], in generating HSNO, which has been proposed as a key intermediate in intracellular sulfur and nitrogen signaling. Although further investigation is required to fully elucidate the mechanism underlying L-CysNO-induced irreversible CSE inhibition at Cys136 and/or Cys171, our findings suggest that CSE metabolizes L-CysNO, leading to enzyme inactivation during L-CysNO metabolism as illustrated in Figure 9. This phenomenon may have physiological significance if the irreversible inhibition observed in this study is also present under (patho)physiological conditions.

## Data Availability

Data is contained within the article or Supplementary Materials. Further inquiries can be directed to the corresponding author(s).

## Supplementary Materials

The following supporting information can be downloaded at https://www.mdpi.com/article/10.3390/antiox14091113/s1. Figure S1: Preparation of immobilized GST-CSE; Figure S2: Inhibition of CaMKII activity by the metabolic products of the CSE/L-CysNO reaction; Figure S3: Product analysis of CSE with L-CysNO; Figure S4. Effect of Angeli’s salt on CSE activity; Supplementary Table S1: The primer lists used.

## Abbreviations

The following abbreviations are used in this manuscript:

CARS	cysteinyl-tRNA synthetase
CaM	calmodulin
CBS	cystathionine β-synthase
CSE	cystathionine-γ-lyase
Cys-SSH	cysteine hydropersulfide
D-CysNO	D-S-nitrosocysteine
L-CysNO	L-S-nitrosocysteine
DTT	dithiothreitol
GSNO	S-nitrosoglutathione
NO	nitric oxide
PAG	DL-propargylglycine
PAGE	polyacrylamide gel electrophoresis
PLP	pyridoxal 5′-phosphate
SDS	sodium dodecyl sulfate
SNAP,	S-nitroso-N-acetylpenicillamine
SSP4	sulfane sulfur probe 4
TCEP	tris(2-carboxyethyl)phosphine
