# Supplementary material for: The Supersulfide-Producing Activity of Rat Cystathionine γ-Lyase Is Irreversibly Inactivated by L-CysNO but Not by L-GSNO
Source: Antioxidants (Basel). 2025 Sep 13;14(9):1113. doi: 10.3390/antiox14091113 (PMC12466479; doi:10.3390/antiox14091113)
Supplement: Supplementary file 1 [file antioxidants-14-01113-s001.zip › antioxidants-3775034-supplementary.pdf]

Supplementary Information

The Supersulfide-Producing Activity of Rat Cystathionine γ-lyase Is Irreversibly Inactivated by L-CysNO but Not by L-GSNO

Shoma Araki [1], Tsuyoshi Takata [2], Sunghyeon Yoon [1], Shingo Kasamatsu [3], Hideshi Ihara [3], Hidehiko Nakagawa [4], Takaaki Akaike [2], Yukihiro Tsuchiya [1,*], and Yasuo Watanabe [1,*]

1 Department of Pharmacology, Showa Pharmaceutical University, Machida 194-8543, Japan; yakuri.araki@gmail.com (S.A.); yoonsh@ac.shoyaku.ac.jp (S.Y.)

2 Department of Redox Molecular Medicine, Tohoku University Graduate School of Medicine,

Sendai 980-8575, Japan; tsuyoshi.takata.a5@tohoku.ac.jp (T.T.); takaike@med.tohoku.ac.jp (T.A.)

3 Department of Biological Chemistry, Graduate School of Science, Osaka Metropolitan University, Sakai 599-8531, Japan; kasamatsu@omu.ac.jp (S.K.); iharah@omu.ac.jp (H.I.)

4 Graduate School of Pharmaceutical Sciences, Nagoya City University, Nagoya 467-8603, Japan; deco@phar.nagoya-cu.ac.jp

* Correspondence: yatsuchi@ac.shoyaku.ac.jp (Y.T.); yasuwata@ac.shoyaku.ac.jp (Y.W.);

Tel.: +81-42-721-1511 (ext. 2121) (Y.T.); +81-42-721-1549 (Y.W.)

SUPPLEMENTARY METHODS

Kinase assay

The total activity of CaMKII (42 nM) was measured for 3 min at 30 °C in 40 mM HEPES/NaOH (pH 7.5), 10 mM Mg $(CH_3COO)_2$, 1 mM $CaCl_2$, 1 μM CaM, 10 mM [γ-^{32}P] ATP, 100 μg/ml BSA, and 50 mM synthetic peptide Syntide-2 (PLARTLSVAGLPCKK). ^{32}P incorporation was determined by spotting 15 ml aliquots of the assay mixture on to Whatman P-81 phosphocellulose paper, followed by washing in 75 mM phosphoric acid.

Nitroxyl detection by P-Rhod fluorescence.

Nitroxyl (HNO) produced by CSE from L-CysNO was measured by P-Rhod fluorescence [1]. Recombinant CSEs (50 μg/mL) were incubated in 20 mM HEPES (pH 7.5), containing 50 μM PLP with buffer alone or 1 mM L-CysNO for 10 min at 30 °C. Samples of interest were reacted with 10 μM P-Rhod in 20 mM Tris-HCl (pH 7.4) in the presence of 50 μM cetyltrimethylammonium bromide in the dark for 10 min at room temperature. Fluorescence intensities of the resultant solutions were determined using a microplate reader (BioTek Synergy HTX Multimode Reader, Agilent Technologies, Inc., Santa Clara, CA, U.S.A.) with an excitation wavelength of 485/20 nm and an emission wavelength of 528/20 nm.

SUPPLEMENTARY FIGURE LEGENDS

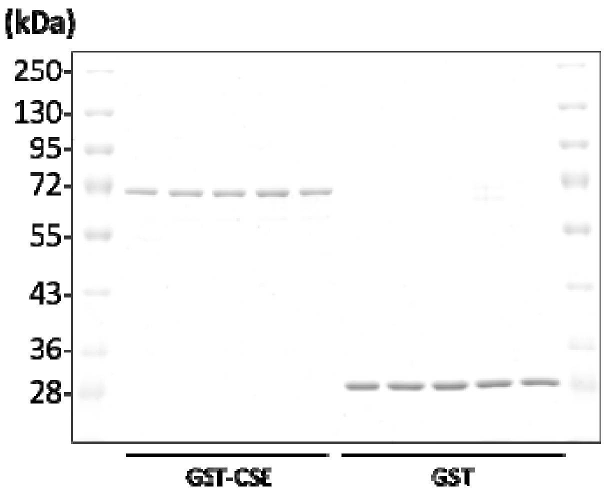

Figure S1. Preparation of immobilized GST-CSE. GST-CSE or GST empty vector was expressed in E. coli (DH5α) using pGEX-6P and immobilized on GSH-agarose (50% slurry) from 2 mg/mL lysate in 50 mM HEPES (pH 7.5) containing 150 mM NaCl, 0.2 mM EDTA, 0.2 mM EGTA, 0.5 mM PMSF, 1 mM DTT, and 10 mg/mL lysozyme (FUJIFILM Wako Pure Chemical Corp., Osaka, Japan). The resin was washed three times with 50 mM HEPES (pH 7.5) at 4 °C. Twenty microliters of resin (50% slurry) was used per assay, and bound proteins were analyzed by SDS-PAGE.

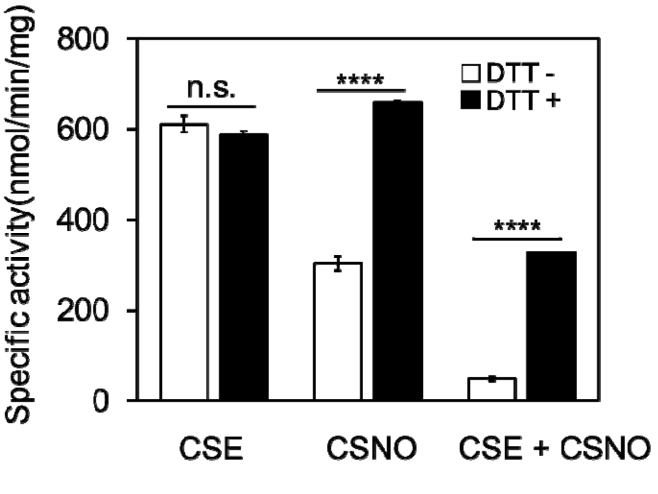

Figure S2. Inhibition of CaMKII activity by the metabolic products of the CSE/L-CysNO reaction.

Purified CaMKII (574 nM) was pre-incubated with CSE (50 μg/ml), L-CysNO (0.3 mM; CSNO), or a combination of CSE (50 μg/ml) and L-CysNO (0.3 mM; CSE+CSNO) in 250 mM HEPES/NaOH buffer (pH 7.5) containing 50 mM PLP and 0.5 mM EDTA at 30 °C for 30 min Following incubation, the total activity of CaMKII (42 nM, adjusted to an equivalent amount) was measured for 3 min at 30 °C in a reaction buffer containing 40 mM HEPES/NaOH (pH 7.5), 10 mM magnesium acetate, 1 mM $CaCl_2$, 1 μM calmodulin, 10 μM $[\gamma\text{-}^{32}P]$ATP, 100 μg/ml BSA, and 50 mM of the synthetic peptide Syntide-2 (PLARTLSVAGLPCKK). ^{32}P incorporation was assessed by spotting 15 μl aliquots of the reaction mixture onto Whatman P-81 phosphocellulose paper, followed by washing in 75 mM phosphoric acid. For the DTT conditions, samples were additionally incubated with 20 mM DTT at 30 °C for 20 min prior to assessment. Results are the mean ± SE of four independent experiments. ****$p < 0.0001$ when compared with control of each sample without DTT. n.s.; not significant.

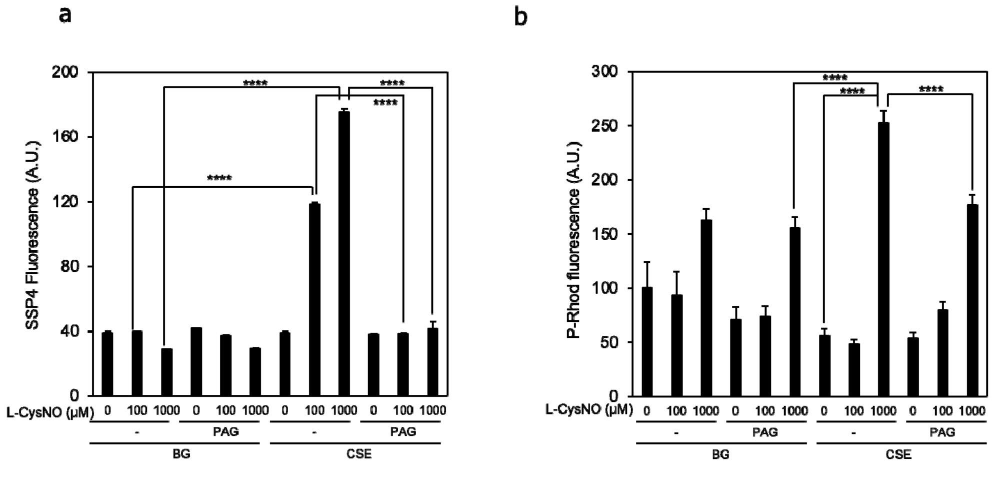

Figure S3. Product analysis of CSE with L-CysNO. (a) The production of CysSSH. The indicated

concentrations of L-CysNO, in the absence or presence of 1mM PAG were incubated with the buffer alone (BG) or with purified CSE (50 μg/mL) at 30 °C for 30 min in a buffer containing 250 mM Hepes/NaOH (pH 7.5) and 62.5 mM PLP. CysSSH production was then detected using the SSP4 fluorescent probe. (b) The production of HNO. The indicated reagents were incubated with the buffer alone (BG) or with purified CSE (50 μg/mL) at 30 °C for 30 min in a buffer containing 250 mM Hepes/NaOH (pH 7.5) and 62.5 mM PLP. HNO production was then detected using the P-Rhod fluorescent probe. Results are the mean ± SE of four independent experiments. ****p < 0.0001.

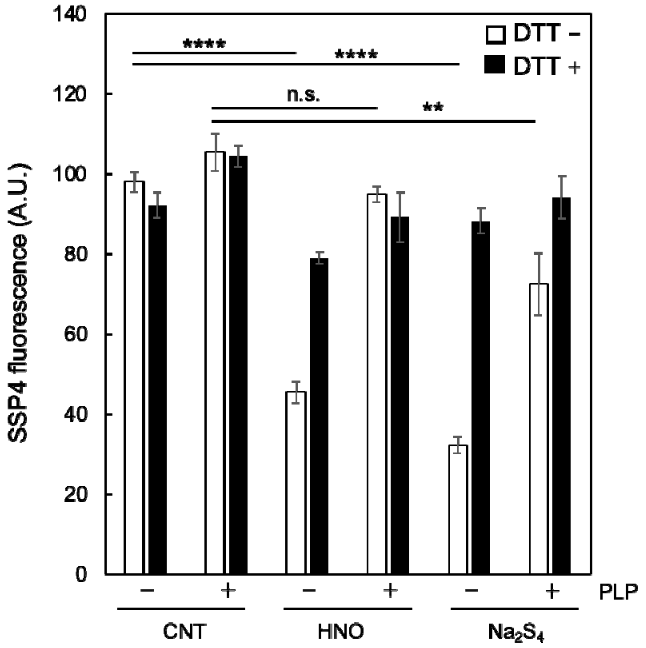

Figure S4. Effect of Angeli's salt on CSE activity. Immobilized GST-CSE was preincubated at 30 °C for 10 min with either buffer alone (CNT), 100 μM Angeli's salt (HNO), or 10 μM Na_2S_4, in the presence or absence of 50 μM PLP. After centrifugation to remove residual Angeli's salt or Na_2S_4, CSE activity was assessed in a reaction mixture containing 50 μM PLP and 1 mM cystine. CysSSH production was then

detected using the SSP4 fluorescent probe. For the DTT condition, samples were additionally incubated with 20 mM DTT at 30 °C for 20 min, followed by centrifugation prior to activity assessment. Buffer alone was used as a blank, and data are presented as increases relative to background values. Results are the mean ± SE of four independent experiments. **** $p < 0.0001$, ** $p < 0.01$, and * $p < 0.05$. n.s.: not significant.

1. Kawai, K.; Ieda, N.; Aizawa, K.; Suzuki, T.; Miyata, N.; Nakagawa, H., A reductant-resistant and metal-free fluorescent probe for nitroxyl applicable to living cells. *J. Am. Chem. Soc.* **2013,** 135, (34), 12690-6.

Supplementary Table S1. The primer lists used.

XhoI_ratCSE	AAAACTCGAGATGCAGAAGGACG
ratCSE_HA_NotI	AAAAGCGGCCGCTTAGGCATAG
CSE_C69V_F	CTGGAAATCCGACGAGGAATGTCTTGGAAAAAGCAGTGGC
CSE_C69V_R	GCCACTGCTTTTTCCAAGACATTCCTCGTCGGATTTCCAG
CSE_C83V_F	GGATGGGGCAAAGCACGTTTTGACCTTCGCCTCG
CSE_C83V_R	CGAGGCGAAGGTCAAAACGTGCTTTGCCCCATCC
CSE_C108V_F	CCATCTTTTAAAAGCAGGAGATGAAGTCATTGTCATGGATGAAGTGTATG
CSE_C108V_R	CATACACTTCATCCATGACAATGACTTCATCTCCTGCTTTTAAAAGATGG
CSE_C136V_F	GGACTGAAGATTTCTTTTGTGGATGTTTCCAAAACCAAATTGCTGGAG
CSE_C136V_R	CTCCAGCAATTTGGTTTTGGAAACATCCACAAAAGAAATCTTCAGTCC
CSE_C171V_F	GTTGGCCGACATCAAAGCCGTCGCACAAATTGTCCACAAA
CSE_C171V_R	TTTGTGGACAATTTGTGCGACGGCTTTGATGTCGGCCAAC

CSE_C205V_F	CTTTGGCTCTGGGTGCTGATATTGTTATGTGTTCTGCCACAAA
CSE_C205V_R	TTTGTGGCAGAACACATAACAATATCAGCACCCAGAGCCAAAG
CSE_C207V_F	GCTCTGGGTGCTGATATTTGTATGGTTTCTGCCACAAAATACATGAA
CSE_C207V_R	TTCATGTATTTTGTGGCAGAAACCATACAAATATCAGCACCCAGAGC
CSE_C251V_F	GTTCCTTCTCCTTTCGATGTTTACCTCTGCTGCCGAGG
CSE_C251V_R	CCTCGGCAGCAGAGGTAAACATCGAAAGGAGAAGGAAC
CSE_C254V_F	CCTTTCGATTGTTACCTCGTCTGCCGAGGCCTGAAGAC
CSE_C254V_R	GTCTTCAGGCCTCGGCAGACGAGGTAACAATCGAAAGG
CSE_C255V_F	TCGATTGTTACCTCTGCGTCCGAGGCCTGAAGACAC
CSE_C255V_R	GTGTCTTCAGGCCTCGGACGCAGAGGTAACAATCGA
CSE_C306V_F	CGCCAAACGTCAGGTCACGGGCTGCCCC
CSE_C306V_R	GGGGCAGCCCGTGACCTGACGTTTGGCG
CSE_C309V_F	TCAGTGCACGGGCGTCCCCGGGATGGTC
CSE_C309V_R	GACCATCCCGGGGACGCCCGTGCACTGA
